# Melatonin protects against apoptosis of megakaryocytic cells via its receptors and the AKT/mitochondrial/caspase pathway

**DOI:** 10.18632/aging.103483

**Published:** 2020-07-10

**Authors:** Mo Yang, Liang Li, Shichao Chen, Suyi Li, Bo Wang, Changhua Zhang, Youpeng Chen, Liuming Yang, Hongwu Xin, Chun Chen, Xiaojun Xu, Qing Zhang, Yulong He, Jieyu Ye

**Affiliations:** 1 The Seventh Affiliated Hospital, Sun Yat-Sen University, Shenzhen, Guangdong, China; 2 Nanfang Hospital, Southern Medical University, Guangzhou, China; 3 Lianjiang People’s Hospital, Lianjiang, Guangdong, China; 4 State Key Laboratory of Biocontrol, School of Life Sciences, Sun Yat-sen University, Guangzhou, China; 5 The Seventh Affiliated Hospital, Sun Yat-Sen University, Shenzhen, Guangdong, China.

**Keywords:** melatonin, apoptosis, thrombopoiesis, megakaryocytic cells, platelets

## Abstract

Clinical studies have shown that melatonin lowers the frequency of thrombocytopenia in patients with cancer undergoing radiotherapy or chemotherapy. Here, we investigated the mechanisms by which melatonin promotes platelet formation and survival. Our results show that melatonin exerted protective effects on serum-free induced apoptosis of CHRF megakaryocytes (MKs). Melatonin promoted the formation of MK colony forming units (CFUs) in a dose-dependent manner. Using doxorubicin-treated CHRF cells, we found that melatonin rescued G2/M cell cycle arrest and cell apoptosis induced by doxorubicin. The expression of p-AKT was increased by melatonin treatment, an effect that was abolished by melatonin receptor blocker. In addition, we demonstrated that melatonin enhanced the recovery of platelets in an irradiated mouse model. Megakaryopoiesis was largely preserved in melatonin-treated mice. We obtained the same results *in vivo* from bone marrow histology and CFU-MK formation assays. Melatonin may exert these protective effects by directly stimulating megakaryopoiesis and inhibiting megakaryocyte apoptosis through activation of its receptors and AKT signaling.

## INTRODUCTION

Melatonin is a product generated from serotonin via N-acetyltransferase and 5-hydroxyindole-O-methyltransferase [1]. In humans, it is abundant in the pineal gland and is released into the bloodstream in a cycle that follows the circadian rhythm. There are three melatonin receptor subtypes: MT_1_, MT_2_ and MT_3_ [2]. MT_1_ and MT_2_ belong to the G-protein coupled receptors family and are able to launch multiple signaling cascades [3]. While we know that MT_3_ is a detoxification enzyme [4], the signal transductions it might initiate remain unidentified.

Melatonin participates in a wide range of physiological processes, such as circadian rhythm and cardiac vessel regulation, anti-oncogenesis, and retinal physiology [5–8]. Therefore, it may have many potential applications in treating various diseases. Clinical research suggests that short-term use of melatonin is safe, even in extreme doses [9]. Recently, bone marrow (BM) cells were found to express melatonin and methyltransferase, which had been considered to be strictly present in pineal gland [10]. Melatonin has been intensively studied in patients with thrombocytopenia since 1995. Studies found that melatonin potently increased platelet count in thrombocytopenic patients [11], especially in those with chemotherapy- and radiotherapy-induced thrombocytopenia [12]. Patients with melatonin administration developed less thrombocytopenia and tumor regression rates were higher compared to those who received chemotherapy alone [13]. However, the exact mechanisms by which melatonin protects against thrombopoiesis and how it interacts with megakaryocytes (MK)/platelets is still unclear.

In this study, we aimed to explore the effects of melatonin on megakaryopoiesis and thrombopoiesis, and their respective underlying mechanisms, in a myelosuppression mouse model. We also investigated melatonin’s anti-apoptotic role in CHRF cells as well as its possible role in treating chemotherapy-induced thrombocytopenia (CIT).

## RESULTS

### Melatonin enhanced the proliferation of CHRF cells

**Figure 1:**
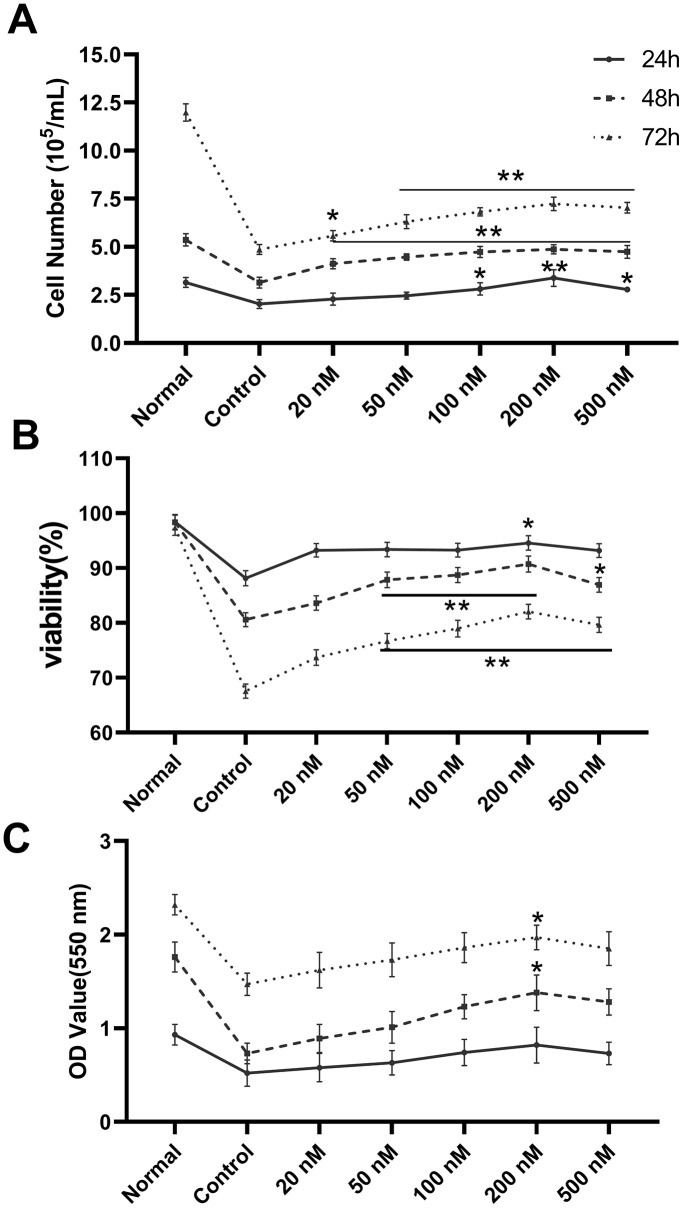
**Melatonin treatment enhances the proliferation of CHRF cells.** The maximum action concentration was 200 nM (n=6). Cell proliferation was assessed by (**A**) cell counts, (**B**) survival rate and (**C**) XTT assay at 24 h, 48 h and 72 h after administration of melatonin treatment. Normal, CHRF cells incubated with 10% FBS; Control, CHRF cells incubated with 0.5% FBS. Two-way ANOVA (with a Tukey multiple comparison test) was employed to test for significance. * p < 0.05, ** p < 0.01 vs control group.

Cell count, survival rate, and XTT assays were used to examine the proliferative effect of melatonin on megakaryopoiesis. We observed increased proliferation under melatonin treatment compared to serum-depleted controls in CHRF cells (Figure 1). Consistent results were obtained from cell counts (Figure 1A), survival rate (Figure 1B), and XTT assays (Figure 1C). The optimal action concentration was 200 nM (n=6).

### Melatonin exerted anti-apoptotic effects in CHRF cells

**Figure 2:**
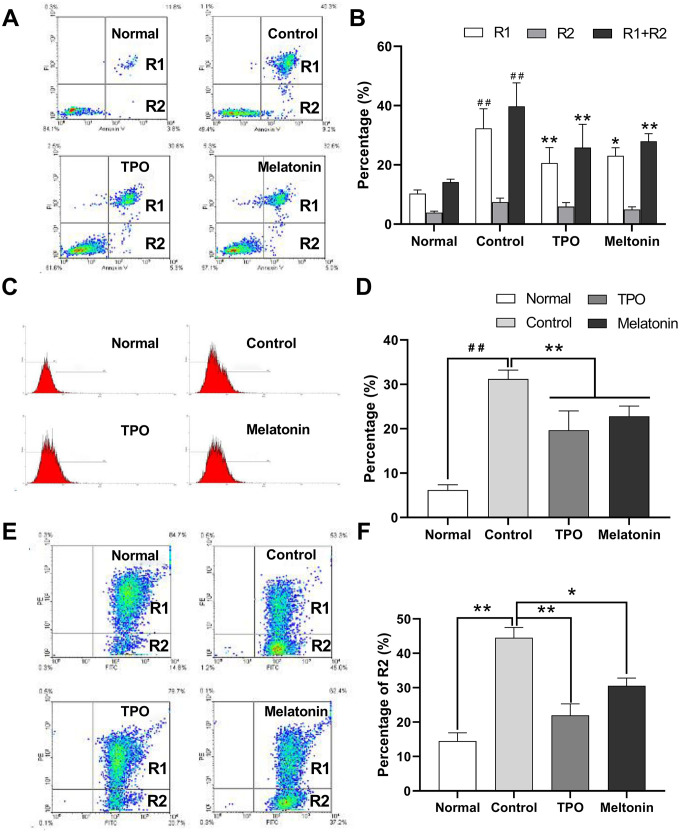
**Melatonin significantly suppresses the apoptosis of nutrition-depleted CHRF cells.** Cells were treated with saline (control), melatonin (200 nM) or TPO (positive control, 100 ng/mL) for 72 h. CHRF cells were harvested and subject to assays to measure the levels of (**A**) Annexin/PI (n=4), (**C**) activation of Caspase-3 (n=3) or (**E**) polarized mitochondrial membrane potential (JC-1, n=3). (**B**), (**D**, **F**) were the statistical analyses of (**A**, **C**, **E**), separately. Normal, CHRF cells incubated with 10% FBS; Control, CHRF cells incubated with 0.5% FBS. One-way ANOVA or Two-way ANOVA (with a Tukey multiple comparison test) was employed to test for significance. # # Compared with normal group, p< 0.01; * p < 0.05, ** p < 0.01.

Nutrition-depleted CHRF cells were treated with saline, melatonin or TPO (positive control), and apoptosis was measured by flow cytometry using AnnexinV/PI, caspase-3 and JC-1 assays. Late apoptotic and necrotic cells (Annexin V positive, PI positive, R2) and total dead cells (Annexin V positive, R1 + R2) were increased in the saline controls compared to the normal nutrition samples (Figure 2A, 2B). Melatonin reduced the proportion of late apoptotic cells as well as total dead cells (Figure 2B). These data indicated that melatonin has an anti-apoptotic effect on megakaryocytes.

Expression of active caspase-3 in serum and cytokine-depleted controls was higher than in normal samples. Addition of melatonin decreased active caspase-3 expression (Figure 2C, 2D). The data suggested that melatonin may exert its anti-apoptotic effects by downregulating caspase-3 activity.

The proportion of cells containing JC-1 monomers (R1) was increased in nutrition-depleted cells compared with that in normal samples (FITC positive, PE negative), indicating a drop in polarized mitochondrial membrane potential (ΔΨ) and an increase in the number of apoptotic cells. Treatment with melatonin reduced the population of apoptotic cells (Figure 2E, 2F), suggesting that the anti-apoptotic effects of melatonin may be mediated by the intrinsic mitochondrial pathway.

### Melatonin enhanced proliferation on doxorubicin-treated MK cells

**Figure 3:**
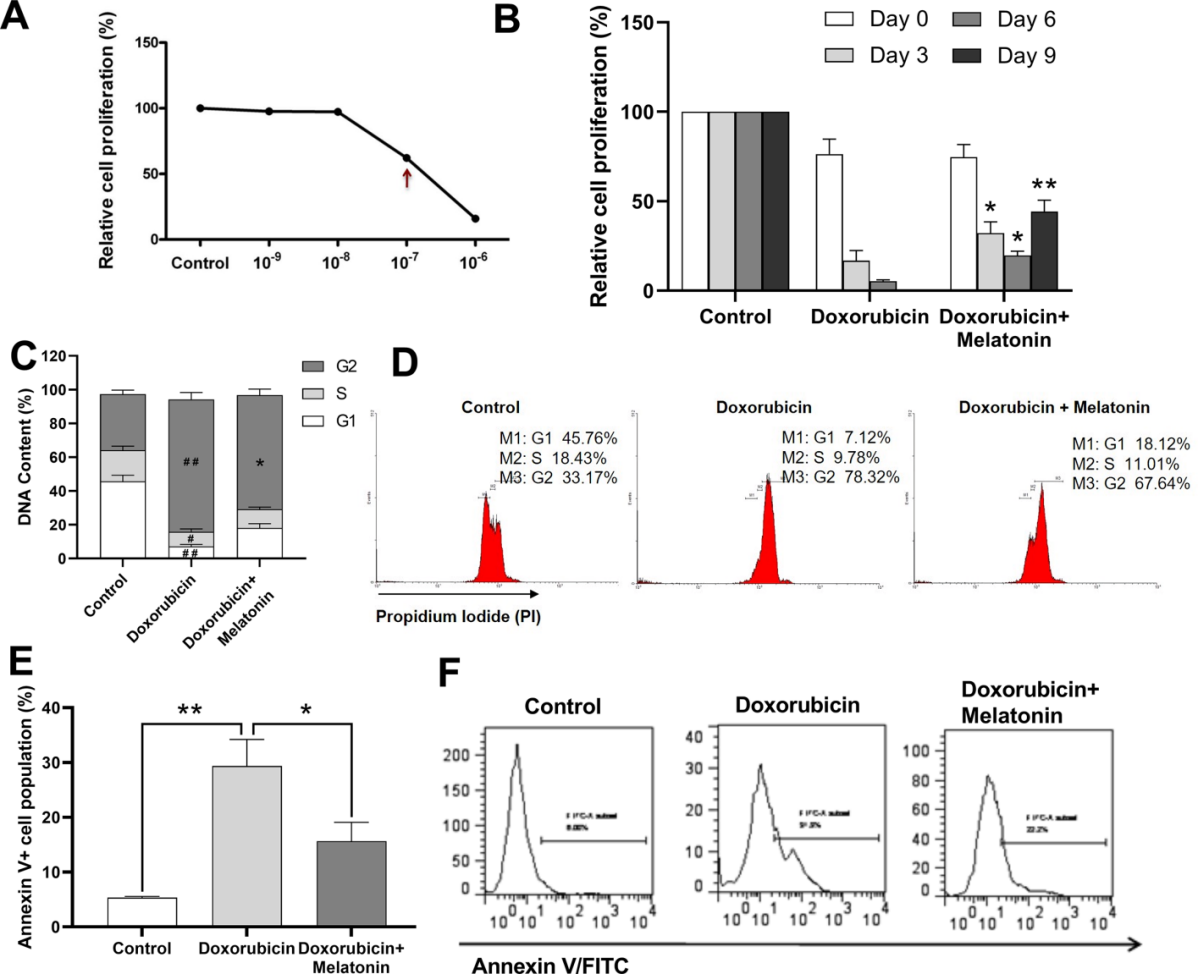
**Melatonin enhances proliferation on doxorubicin-treated CHRF cells and repairs G2/M cell cycle arrest induced by doxorubicin.** (**A**) Relative cells proliferation treated by different concentration (10^-9^ M~10^-7^ M) of doxorubicin for 24 h. (**B**) Proliferation analysis of doxorubicin-treated CHRF cells with or without melatonin (200 nM) on days 0, 3, 6 and 9, (n=3). (**C**) Cell cycle analysis of doxorubicin-treated CHRF cells with or without melatonin (200 nM) for four days (n=4). (**D**) Cell cycle assessed by flow cytometry (n=4). (**E**) Cell apoptosis analysis of doxorubicin-treated CHRF cells with or without melatonin (200 nM) for, n=3. (**F**) Detection of Annexin V by flow cytometry, n=3. One-way ANOVA or Two-way ANOVA (with a Tukey multiple comparison test) was employed to test for significance. #Compared with control group, p< 0.05, # # p< 0.01; * compared with doxorubicin-treat group, p< 0.05, ** p< 0.01.

Figure 3A illustrated that the IC_50_ of doxorubicin on CHRF cells was at around 10^-7^ M. Melatonin was added to doxorubicin-treated CHRF cells to test whether it could rescue them from toxicity. On Day 3, cell proliferation remarkably decreased in the doxorubicin-treated group, and this toxic effect continued and worsened by Day 6 and Day 9 compared with untreated controls. Addition of melatonin reversed the damage and stimulated cell proliferation from Day 3. The rescue effect persisted and became more obvious on Day 6 and Day 9 (Figure 3B), suggesting that melatonin had a protective effect on chemotherapeutic drug-treated MK cells.

### Melatonin repaired G2/M cell cycle arrest induced by doxorubicin

To investigate the potential mechanism by which melatonin rescued doxorubicin-induced toxicity, we used cell cycle assay to measure DNA contents in doxorubicin-treated CHRF cells with or without melatonin treatment. The G_2_ phase in doxorubicin-treated cells increased while G_1_ and S phase decreased, relative to untreated control (Figure 3C), which was an indication of cells undergoing G2/M cell cycle arrest. Upon melatonin treatment, the proportion of cells in G_2_ phase was reduced (*P*=0.0125) and that of cells in G_1_ and S phase was increased (Figure 3C, 3D). This suggested that melatonin can revert G2/M cell cycle arrest, pushing the cells into M phase to start mitosis.

### Melatonin rescued doxorubicin-induced cell apoptosis

In order to further explore the effects of altered cell cycle progression, we analyzed apoptosis in CHRF cells using Annexin V assay. We observed upregulation in the expression of Annexin V/FITC in doxorubicin-treated cells (Figure 3E, 3F) while melatonin treatment attenuated this effect (Figure 3E, 3F). Our data indicated that melatonin reduced doxorubicin-initiated apoptosis, consistent with its role in repairing G2/M cell cycle arrest.

### Detection of melatonin receptors

**Figure 4:**
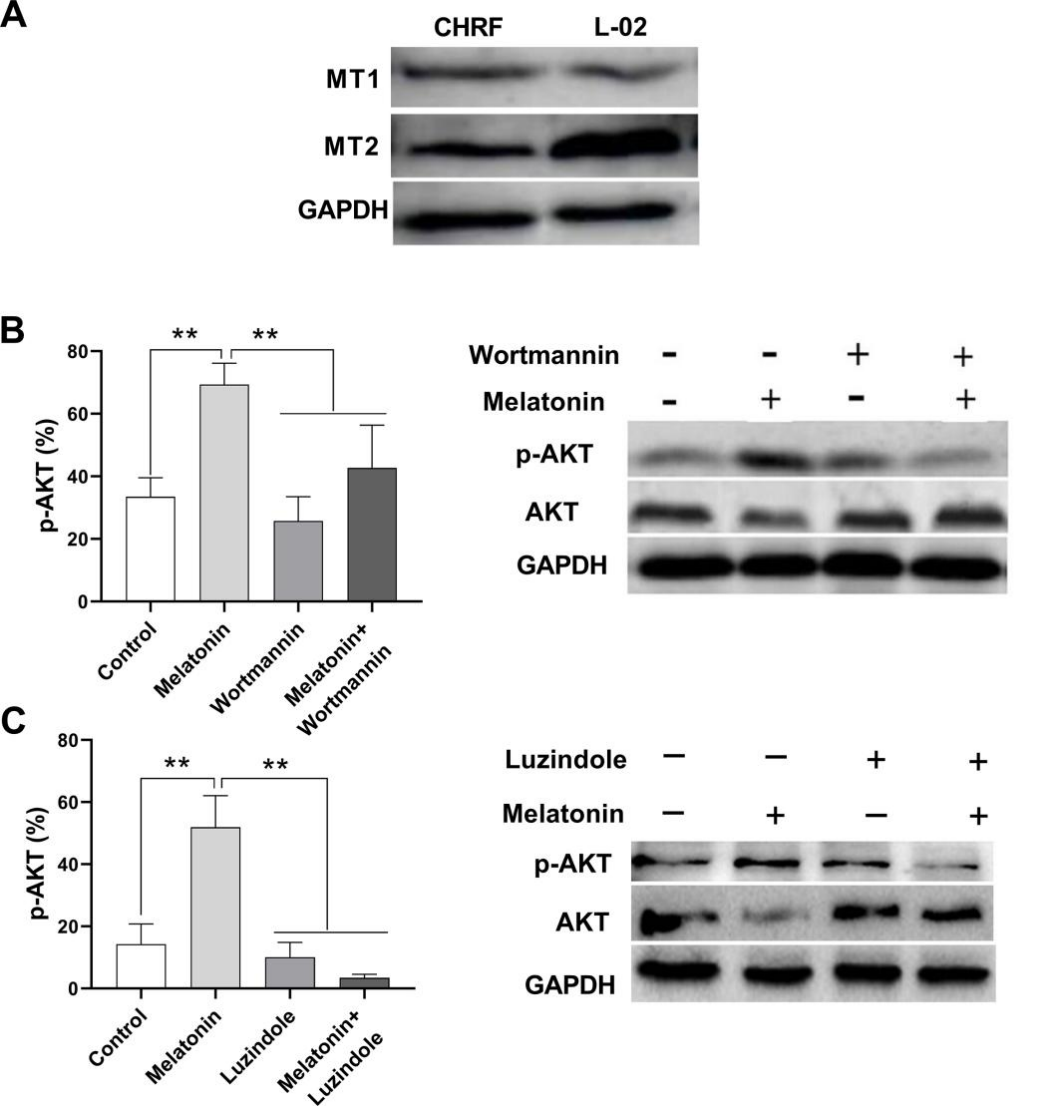
**Melatonin increases the expression of p-AKT and this effect could be suppressed by Wortmannin and luzindole.** Wortmannin is a non-specific, covalent inhibitor of PI3K. luzindole acts as a selective melatonin receptor antagonist for the MT2 receptor. A 30 min preincubation step with the PI3K inhibitor Wortmannin (100 nM) or a 60 min preincubation step with the MT2 receptor antagonist Luzindole (1 μM) was included before melatonin stimulation. (**A**) Melatonin receptors MT1 and MT2 were detected in megakaryocytic CHRF cells through Western Blot. L-02 cells was the positive control. P-AKT was also detected by Western Blot. Cells were treated with (**B**) melatonin (200 nM), wortmannin (100 nM) or melatonin+wortmannin and (**C**) melatonin (200 nM), luzindole (1 μM) or melatonin+luzindole. Two-way ANOVA (with a Tukey multiple comparison test) was employed to test for significance. * p< 0.05, ** p< 0.01, n=5.

Liver cells were shown to express MT1 and MT2 receptors [14]; thus, here we used a normal liver cell line, L-02, as a positive control. We detected MT1 and MT2 receptors in megakaryocytic CHRF cells by Western Blot (Figure 4A).

### Effect of melatonin on p-AKT expression on CHRF cells

The expression of phosphor-AKT (p-AKT) was enhanced by melatonin treatment in CHRF cells compared to untreated samples. On the other hand, addition of wortmannin (a non-specific, covalent inhibitor of PI3K) and luzindole (a selective melatonin receptor antagonist for MT_2_ receptor) suppressed p-AKT expression (Figure 4B, 4C).

### Melatonin promoted the formation of murine colony forming units for megakaryocytes and fibroblasts *in vitro* as well as the proliferation of CHRF cells

**Figure 5:**
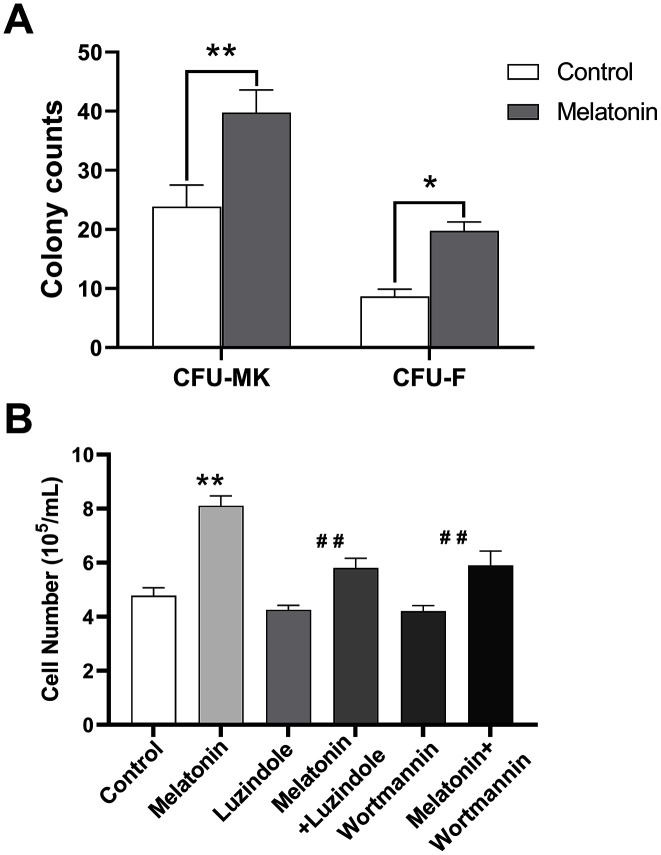
**Effect of melatonin on CFU-MK, CFU-F and CHRF cells.** Bone marrow cells were seeded with or without melatonin (200 nM) for nine days and identified by Giemsa staining. CHRF cells were treated with melatonin (200 nM), wortmannin (100 nM), melatonin+wortmannin, luzindole (1 μM) and melatonin+luzindole. A 30 min preincubation step with the PI3K inhibitor Wortmannin (100 nM) or a 60 min preincubation step with the MT2 receptor antagonist Luzindole (1 μM) was included before melatonin stimulation. (**A**) Melatonin promotes the formation of murine CFU-MK and CFU-F. (**B**) Melatonin has a promoting effect on the proliferation of CHRF cells, adding wortmannin and luzindole can inhibit this effect. Two-way ANOVA (with a Tukey multiple comparison test) was employed to test for significance. * p< 0.05, ** p< 0.01, n=4. CFU-MK, colony- forming unit-megakaryocyte; CFU-F, colony forming unit- fibroblast.

We further analyzed the effect of melatonin on colony-forming-unit formation for murine bone marrow cells. Our results showed that melatonin treatment simulated CFU-megakaryocyte (CFU-MK) and CFU-fibroblast (CFU-F) formation compared to the control group (Figure 5A). In addition, melatonin promoted the proliferation of CHRF cells while adding wortmannin and luzindole inhibited this effect (Figure 5B).

### Effect of melatonin on blood cell counts in mouse model

**Figure 6:**
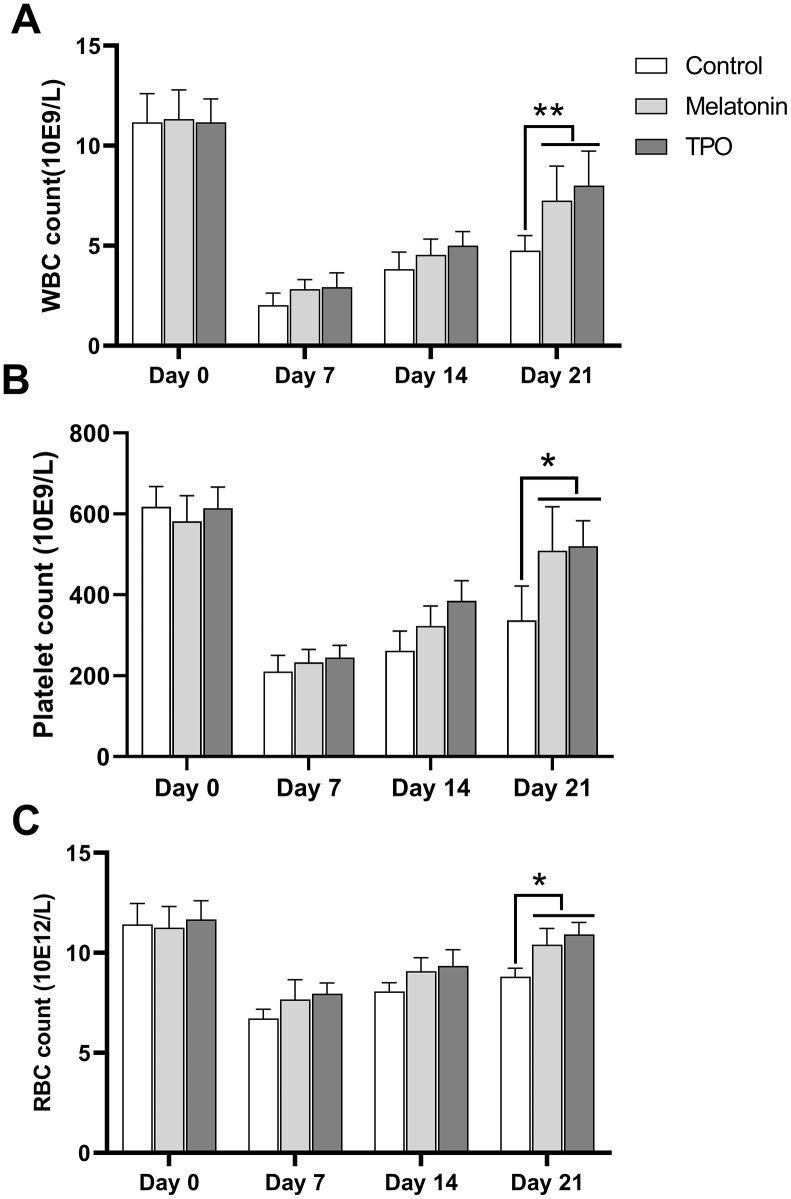
**Melatonin increases peripheral blood cell counts in the radiation-induced myelosuppression mouse.** Mice were treated with melatonin (10 mg/kg/day) or TPO (positive control, 1 μg/kg/day) by injecting intraperitoneally. The injections were performed once a day starting from the day of irradiation. (**A**) white blood cells count. (**B**) Platelets count. (**C**) red blood cells count. The effect of melatonin was similar to TPO. Two-way ANOVA (with a Tukey multiple comparison test) was employed to test for significance. * p< 0.05, ** p< 0.01, n=6. WBC, white blood cells; RBC, red blood cells.

At Day 0, the basal numbers of peripheral white blood cell (WBC) were approximated to 11×10^9^/L and decreased after irradiation to the nadir count of 2-3×10^9^/L at day 7. The cells began to recover from Day 14. Both melatonin and TPO had stimulating effects on WBC recovery (Figure 6A). The melatonin-treated group showed better recovery as compared to the saline control group at Day 21. Peripheral platelets in experimental mice decreased after irradiation from ~600×10^9^/L at Day 0 to the nadir counts of 200×10^9^/L at Day 7 and recovered gradually (Figure 6B). The melatonin-treated group showed better recovery at Day 21. Similarly, the peripheral RBC decreased following irradiation, with the nadir appearing at Day 7 and started increasing thereafter. Compared to the saline control group, melatonin treatment increased the number of RBC on Day 21 (Figure 6C). Our results demonstrated that melatonin has protective effects on peripheral blood cell recovery, similar to the effect of TPO.

### Effect of melatonin on total body weight and organ weight

All mice lost weight (about 5-10%) after irradiation at Day 7, then recovered gradually (Table 1). Total body weight of mice under different treatments did not show any differences. To make the assessment more comparable, the organ weight of liver, spleen and kidney from animals under different treatments were normalized to their body weight and expressed as the ratio of organ weight to body weight (Table 2). There were again no differences in the ratio between the different groups (Table 3).

**Table 1:** The effect of melatonin on body weight (n=6).

	**Normal**	**Control**	**Melatonin**	**TPO**
Day 0	26.83±0.63	27.11±1.03	26.24±0.83	27.58±0.64
Day 7	27.48±0.75	26.46±0.92	25.85±0.58	26.27±0.41
Day 14	27.83±0.39	27.32±0.93	26.35±0.72	27.16±0.38
Day 21	28.00±0.45	28.17±1.05	26.90±0.83	28.00±0.52

TPO, thrombopoietin.

**Table 2:** The effect of melatonin on organ weight (n=6).

	**Normal**	**Control**	**Melatonin**	**TPO**
Liver	1.34±0.045	1.45±0.084	1.26±0.040	1.44±0.059
Kidney	0.49±0.015	0.48±0.046	0.45±0.023	0.53±0.014
Spleen	0.11±0.009	0.13±0.022	0.11±0.016	0.14±0.025

TPO, thrombopoietin.

**Table 3:** The effect of melatonin on organ weight/body weight (n=6).

	**Normal**	**Control**	**Melatonin**	**TPO**
Liver	0.048±0.0013	0.050±0.0019	0.047±0.0018	0.051±0.0016
Kidney	0.018±0.0003	0.017±0.0013	0.017±0.0011	0.019±0.0004
Spleen	0.004±0.0004	0.0045±0.0008	0.004±0.0004	0.005±0.0009

TPO, thrombopoietin.

### Effect of melatonin on bone marrow histology

**Figure 7:**
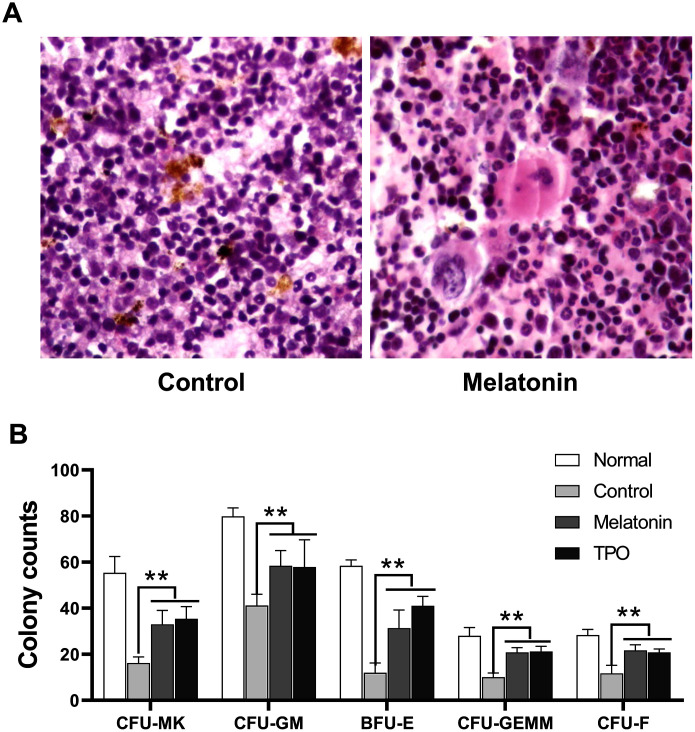
**Melatonin promotes the formation of CFU.** The number of CFUs was scored after melatonin (100 nM) or TPO (positive control, 50 ng/mL) treatments for seven days (n=6). (**A**) Bone marrow histology of Giemsa staining. (**B**) Statistic analysis of CFUs. Two-way ANOVA (with a Tukey multiple comparison test) was employed to test for significance (n=6). ** p< 0.01. CFU-MK, colony- forming unit-megakaryocyte; CFU-GM, colony-forming unit-granulocyte macrophage; BFU/CFU-E, burst-forming unit/colony-forming unit-erythroid; CFU-GEMM, colony-forming unit-mixed.

Bone marrow histological examination was performed on Day 21 after sacrifice. Hematopoiesis in irradiated control samples was largely suppressed with decreased numbers in total cells, especially the cells in megakaryocytic and granulocytic lineages. There was also an increase in the number of necrotic and apoptotic cells compared to normal mouse controls without irradiation (Figure 7A). Hematopoiesis was largely preserved in the melatonin and TPO-treated groups as bone marrow hyperplasic was observed in these mice. The numbers of megakaryocytes and their progenitors were higher than those in control mice, which showed values much closer to normal. In addition, we observed a reduction of apoptotic cells in the melatonin-treated samples (Figure 7A).

### Melatonin promoted the formation of bone marrow CFUs

Results from our murine CFU assays suggested that melatonin enhanced the formation of CFU-MK, CFU-GM, BFU-E, CFU-GEMM and CFU-F as compared with controls (Figure 7B). Consistent with our bone marrow histology results, these CFUs data suggested that melatonin enhanced the recovery of hematopoietic stem and progenitor cells.

## DISCUSSION

Our present study reported that melatonin promoted peripheral platelet recovery in a radiation-induced myelosuppression mouse model, and that exposure of MK cells to melatonin reduced doxorubicin-induced toxicity by reversing G2/M phase cell cycle arrest, thereby increasing their proliferation (Figure 3A–3D). Results from our annexin V assays further demonstrated that melatonin rescued doxorubicin-treated MKs by reducing cell apoptosis (Figure 3E, 3F). Considering its safety and non-tumorigenicity, melatonin may be used as a new thrombopoietic drug to treat thrombocytopenia patients undergoing radio/chemotherapy.

G2/M is one of two cell cycle checkpoints used to monitor, verify, and repair DNA damage [15, 16]. Cells that fail to pass this checkpoint present DNA accumulation in G_2_ phase and a reduction in M phase, which is termed “G2/M arrest” [17], which precludes mitosis and thereby inhibits proliferation.

DNA damage is involved in doxorubicin-induced cell toxicity [18]. Doxorubicin is an anthracycline antibiotic widely used in cancer chemotherapy [19] and is known to induce cell cycle G2/M phase arrest and cell apoptosis in cancer cells and normal cells [20]. The most accepted mechanism is that doxorubicin intercalates into the DNA to induce DNA oxidative damage by producing reactive oxygen species (ROS) [21]. This destructive effect has also been found in megakaryocytes (MKs) and platelets, as chemotherapy-induced thrombocytopenia (CIT) was reported to be one of the most common side effects induced by doxorubicin therapy [22]. Melatonin effectively attenuates the oxidative damage caused by various toxins [23–25] and protects various tissues against doxorubicin damage [26–28]. Concomitant doxorubicin and melatonin treatment did not affect the latter component’s antitumor effectiveness [29].

We measured the expression of melatonin receptors MT1 and MT2 in CHRF cells as well that of p-AKT, which was enhanced by melatonin. Addition of luzindole, a selective melatonin receptor antagonist, suppressed p-AKT expression, suggesting that melatonin works through its receptor. We obtained the same results when the cells were treated with wortmannin, a fungal metabolite that acts as a selective inhibitor of PI3K [30]. In previous studies, AKT was found to participate in the transduction of anti-apoptotic signals in a variety of cells [31], and to mediate anti-apoptotic processes caused by radiation, chemotherapy, ischemic hypoxia and oxidative damage [31, 32]. AKT is activated by growth factors in a PI3K-dependent manner [33]. This indicates that melatonin activates the PI3K/AKT signaling pathway through melatonin receptors. In addition, treatment with melatonin has been shown to increase the polarized mitochondrial membrane potential and suppressed the activation of caspase-3 that induces cell cycle G2/M phase arrest [34, 35]. Furthermore, melatonin treatment also inhibits the mitochondrial apoptosis induced by caspase-9 activity [36–38]. At the molecular level, OPA1-related mitochondrial fusion/mitophagy, which can be normalized by melatonin, has been observe to reverse excessive mitochondrial fission, promote\ mitochondrial energy metabolism, sustain mitochondrial function, and block cardiomyocyte caspase-9-induced mitochondrial apoptosis [36]. Furthermore, melatonin treatment diminished cytochrome c release from mitochondria and reduced caspase 3 and caspase 9 activation induced by hyperhomocysteinemia in neuronal cells [37]. Additionally, melatonin reduces caspase-9 and -3 activities induced by increases in [Ca(2+)] (c) in human leukocytes, which are produced through the inhibition of both mPTP and Bax activation [39]. In summary, the results of our study here suggest that the anti-apoptotic effect of melatonin may be mediated by PI3K/AKT through an intrinsic mitochondrial pathway in a caspase-dependent manner.

Numerous studies have examined the effects of melatonin in the context of various cancers and reported the enhanced efficacy of chemo/radiotherapy in combination with melatonin [40]. The combinations of everolimus plus melatonin and barasertib plus melatonin exhibited impressive synergistic cytotoxic effects on leukemia lymphocytes but did not affect the viability of normal lymphocytes [41]. Melatonin acts as a tumor suppressor and antioxidant [42], and strengthens the anti-cancer potential of chemotherapeutic drugs in human colorectal adenocarcinoma HT-29 cells [43], rat pancreatic tumor cell line AR42J [44], and cervical cancer HeLa cells. Consistently, co-stimulation of HeLa cells with any chemotherapeutic agent in the presence of melatonin further increased caspase-3 activation, particularly in CIS- and 5-FU-challenged cells. Likewise, concomitant treatments with melatonin and CIS enhanced the ratio of cells entering mitochondrial apoptosis due to reactive oxygen species (ROS) overproduction, substantially augmented the population of apoptotic cells, and markedly enlarged DNA fragmentation compared to CIS treatment alone [45]. Therefore, melatonin may be useful in the treatment of tumors in association with chemotherapy drugs.

Melatonin is a derivative of serotonin transferred by acetyltransferase and methyltransferase. Previous studies indicated that melatonin was synthesized not strictly within pineal grand, but also in bone marrow (BM) cells [46]. Since MKs [47] and platelets were primary reservoirs for circulating serotonin, bone marrow MKs maybe one of the major sources for BM melatonin. Although our previous studies suggested that serotonin promotes megakaryopoiesis [48], administration of exogenous serotonin could cause behavioral and digestive disorders, and lead to and overproduction of serotonin because of its multi-organ targeting functions. In contrast, oral administration of melatonin exerts an effective and safe thrombopoietic effect in idiopathic thrombocytopenic purpura patients [49]. More excitingly, melatonin potently enhanced platelet production in CIT patients without affecting the efficiency of chemotherapeutic drugs [48]. Therefore, compared with serotonin, melatonin exhibits a much brighter clinical potential to manage thrombocytopenia.

## MATERIALS AND METHODS

### Reagents and cell line

Melatonin was purchased from Sigma (MO, USA) and doxorubicin was purchased from Pharmacia & Upjohn (NJ, USA). Propidium Iodide (PI) was obtained from Invitrogen (NY, USA). CHRF-288-11 was purchased from ATCC (MD, USA) and cultured with IMDM containing 10% FBS in a concentration of 1×10^6^ cells/mL. Medium was changed every three or four days.

### XTT proliferation assay

CHRF cells were divided into Normal (10% FBS), Control (0.5 FBS) and melatonin-treated (20 nM-500 nM) groups. CHRF cells were incubated with or without melatonin in 96-well plates at 37 °C with 5% CO_2_ for 24-72 h. 50 μl of XTT mixture (1μl XTT1 reagent in 50μl XTT2 reagent) (Roche Applied Science, IN, USA) was added into each well and incubated at 37 °C with 5% CO_2_ for 4 h. The absorbance of samples was measured using microplate reader (Model 550, bio-rad, CA, USA) at 550 nm. Cell relative activity was calculated using Excel program.

### Cell viability assay

CHRF cells were divided into Normal (10% FBS), Control (0.5% FBS) and melatonin-treated (20-500 nM) groups. CHRF cells were incubated with or without melatonin in 6-well plates at 37 °C with 5% CO_2_ for 24-72 h. The density of CHRF cells suspension was determined. 0.4% solution of trypan blue in buffered isotonic salt solution was prepared and 0.1 mL of trypan blue stock solution was added to 1 mL of cells. Cells were examined immediately under a CKX53 Inverted Microscope (OLYMPUS, Japan) at low magnification. To calculate the number of viable cells per mL of culture, we use the following formula: % viable cells = [1.00 – (Number of blue cells ÷ Number of total cells)] × 100*.*

### Cell apoptosis assay

***Annexin V/PI apoptotic assay, Caspase-3 assay and JC-1 assay.*** CHRF cells were cultured with or without melatonin for 72 h. Cell were then collected for flow cytometry analysis and the population of apoptotic cells was measured by three complementary methods: AnnexinV-FITC/PI, active caspase-3-PE, and JC-1 (5, 5, 6, 6-tetrachloro-1, 1, 3, 3-Tetraethylbenzimidazolcarbocyanine iodide) according to the manufacturer’s instructions (BD Biosciences, San Diego) [50, 51]. Ten thousand events were acquired for each sample and analyzed by flow cytometry using the Lysis II software (FACScan; BD Pharmingen).

### Cell cycle assay

Doxorubicin was added in CHRF cell culture for 72 h. The drug was washed away by PBS and cells were re-incubated with or without melatonin for another 24 h. The mixtures were then washed by PBS and cells were fixed with 70% cold ethanol overnight at 4 °C. Cell pellets were collected by centrifugation and re-suspended in p*ropidiumiodide* (*PI*) solution containing 50 μg/mL PI and 0.1 mg/mL RNase A for 30 min at room temperature (RT). DNA contents of samples with different treatment were determined by flow cytometry (*BD*™ *LSR II*, BD Bioscience, CA, USA).

### Western blot

For AKT, p-AKT, MT1 and MT2 immunodetection, cells were plated at initial densities of 5.0 × 10^5^ cells in 35-mm diameter plates overnight. A 30 min preincubation step with the PI3K inhibitor Wortmannin (100 nM) or a 60 min preincubation step with the MT_2_ receptor antagonist Luzindole (1 μM) was performed before melatonin stimulation. Cells were treated with melatonin of 200 nM for 45 min. Then, they were rinsed rapidly in ice-cold PBS and lysed in a buffer containing 2% sodium dodecyl sulfate (SDS; Sigma-Aldrich) and 125 mM Tris (pH 6.8) buffer. Lysates were sonicated, and protein was quantified using the DC Protein Assay from Bio-Rad (Hercules, CA). Cell lysates were resolved by SDS-polyacrylamide gel electrophoresis. Membranes were blocked with Tris-buffered saline with Tween 20, 20 mM Tris–HCl (pH 7.4), 150 mM NaCl, and 0.05% Tween 20 containing 5% nonfat dry milk for 1 h at room temperature. Membranes were probed with the appropriate primary antibodies (1:1000; Santa Cruz Biotechnology, Dallas, TX) overnight and subsequently incubated for 1 h with the appropriate peroxidase-conjugated secondary antibodies (1:1000) at the dilutions recommended by the manufacturers [52]. Blots were finally developed with an ECL (Amersham Biosciences, Little Chalfont, UK) Western blotting detection system. Quantitative western bolt analysis of p-AKT was performed using the image analysis software (Image J, NIH, USA), which generates a histogram of pixels according to their color intensity.

### Radiation-induced myelosuppression mouse model

Male seven or eight weeks old Balb/c mice were purchased from Charles River (Yokohama, Japan). Mice were exposed to 3.5 Gy total body irradiation from a ^137^Cs source (Gammacell 1000 Elite Irradiator; MDS Nordion, Kanata, ON, Canada). Eighteen animals were randomly divided into three groups and were injected intraperitoneally (IP) with melatonin (10 mg/kg/day, n=6), or thrombopoietin (TPO, 1 μg/kg/day, n=6) (PeproTech, NJ, USA), which was used as a positive control, or saline (n=6), respectively. The injections were performed once a day starting from the day of irradiation. Mice were sacrificed by cervical dislocation. Peripheral blood samples were collected to perform the cell count for platelets, red blood cells (RBC) and white blood cells (WBC) on days 0, 7, 14 and 21. Mice were sacrificed on day 21 and their bone marrow samples were harvested for colony forming unit (CFU) assays and histology analysis. Ethic permissions for the studies were granted by the Animal Research Welfare Committee of the Sun Yat-Sen University.

### Murine CFU-MK assay

Murine bone marrow cells (2×10^5^) with or without melatonin (100 nM) were plated in 35 mm dishes using the plasma clot culture system, which contained 1% deionized bovine serum albumin (BSA, Sigma, MO, USA), 0.34 mg CaCl_2_, 10% citrated bovine plasma (Sigma, MO, USA) and 50 ng/mL TPO in IMDM with a total volume of 1 mL. Cells were then cultured at 37 °C with 5% CO_2_ for seven days and followed by staining with acetylcholine esterase (AchE). A colony- forming unit-megakaryocyte (CFU-MK) was identified as a cluster of three or more AchE-positive cells.

### Murine CFU-F assay

Mouse bone marrow cells were seeded with or without melatonin in 2 mL of IMDM with 10% FBS in a concentration of 1×10^6^ cells/mL using 35 mm Petri dishes. The cells were maintained in a fully humidified incubator at 37 °C with 5% CO_2_ for nine days. After nine days incubation, cells were washed with PBS and identified by Giemsa staining. A colony forming unit- fibroblast (CFU-F) was defined as a cluster containing 20 or more fibroblasts.

### Murine CFU-Mixed assay

Mouse bone marrow cells (2×10^5^ cells) from three different groups were plated into 35 mm culture dishes in 1% of methylcellulose (Sigma) supplemented with 30% of FCS, 1% of BSA (Gibco), 0.1 mM of β-mercaptoethanol (Sigma), 10 ng/mL of IL-3 (PeproTech, Rocky Hill, NJ), 50 ng/mL of SCF (PeproTech, Rocky Hill, NJ), 3 U/mL of EPO (Calig, Zug, Switzerland), and 10 ng/mL GM-CSF (Sandoz; Basle, Switzerland). The dishes were incubated at 37 °C and 5% CO_2_ in a humidified atmosphere_._ Colony-forming unit-granulocyte macrophage (CFU-GM), burst-forming unit/colony-forming unit-erythroid (BFU/CFU-E), and colony-forming unit-mixed (CFU-GEMM) were scored after seven days.

### Bone marrow histology

Bone marrow samples were frozen in cryomolds and cut into 5 μm sections. The slides were stained with Giemsa staining. Twenty-five random high-power fields from each bone marrow sample were chosen (400×) for mean total cell counts (MTC). Additionally, mean cell counts of three cell lineages, erythroid, granulocytic and megakaryocytic series, were also examined.

### Statistical analysis

All values are expressed as the mean ± standard deviation (SD). Statistical analysis was performed using a two-tailed unpaired Student's *t*-test or analysis of variance (ANOVA) for multiple comparisons. p < 0.05 was considered statistically significant and p < 0.01 as highly significant.
